# Do Sleep Problems Exacerbate the Mental Health Consequences of Discrimination Among Adults?

**DOI:** 10.1097/PSY.0000000000001305

**Published:** 2024-03-25

**Authors:** Thomas E. Fuller-Rowell, Megan M. Zeringue, Ekjyot K. Saini, Tiffany Yip, Mona El-Sheikh

**Affiliations:** Department of Human Development and Family Science, Auburn University, Auburn, Alabama; Department of Psychology, Middle Tennessee State University, Murfreesboro, Tennessee; Department of Human Development and Family Studies, Pennsylvania State University, University Park, Pennsylvania; Department of Psychology, Fordham University, Bronx, New York; Department of Human Development and Family Science, Auburn University, Auburn, Alabama

**Keywords:** perceived discrimination, psychosocial stress, internalizing and externalizing mental health problems, depression, anxiety, within-person sleep variability, sleep actigraphy, sleep duration, social determinants

## Abstract

**Objective:**

An emerging literature suggests that sleep may play an important role in moderating the association between discrimination and mental health problems among adolescents. However, few if any studies have considered this topic among adults. Addressing this knowledge gap, the current study examined multiple sleep parameters as moderating variables in the association between discrimination and mental health problems among adults.

**Methods:**

Participants were 874 adults residing in small towns and semirural contexts within the Southeastern region of the United States (*M*_age_ = 41 years, SD = 7; 57% female; 31% Black, 69% White; 52% income-to-needs < 2). Sleep duration and night-to-night variability in duration were assessed using wrist actigraphy. Established self-report measures were used to assess global sleep problems, experiences of discrimination, and mental health problems (anxiety, depression, and externalizing symptoms).

**Results:**

Experiences of discrimination were associated with more depression, anxiety, and externalizing problems. Two out of three sleep parameters were found to moderate the effects of discrimination on mental health. The association between discrimination and externalizing problems (but not anxiety or depression) was attenuated among those with less night-to-night variability in sleep duration. The associations between discrimination and anxiety and externalizing problems (but not depression) were attenuated among those with fewer global sleep problems. Less variability in sleep duration and fewer global sleep problems were also directly associated with lower levels of depression, anxiety, and externalizing problems.

**Conclusions:**

Greater consistency in sleep duration from night-to-night, and fewer overall sleep problems appear to mitigate risk of mental health problems among adults, particularly in contexts where discrimination is prevalent.

## INTRODUCTION

A large number of studies have examined associations between discrimination and mental health problems; meta-analyses and reviews of the literature suggest that discrimination is associated with higher levels of depression, anxiety, and psychological distress (1,2). Although the majority of the research on discrimination and mental health has focused on internalizing symptoms such as depression and anxiety, several studies have also documented an association between discrimination and externalizing symptoms such as anger or hostility (3–5). A majority of studies considering discrimination and externalizing symptoms have focused on children and adolescents (6,7), with relatively few studies focusing on adults. Reviews of longitudinal and experimental studies suggest a causal relationship with experiences of discrimination leading to increases in psychological distress (8,9). Meta-analyses also indicate that associations between discrimination and mental health or psychological distress appear to be slightly larger in magnitude than associations between discrimination and physical health (1,10,11). In short, accumulating evidence indicates that discrimination is a salient antecedent of mental health problems and suggests the need for further elucidation of modifiable risk and protective factors in this association.

At least three lines of thinking provide the conceptual underpinnings for an association between discrimination and sleep problems. First, discrimination can be considered as a chronic stressor, which leads to dysregulation of the body’s stress response systems. Notably, this affects the hypothalamic-pituitary-adrenal axis and disrupts normal sleep patterns (12,13). Second, discrimination can induce heightened vigilance and psychological distress, which hinders the relaxation needed for healthy sleep initiation and maintenance (14–19). Lastly, the behavioral changes resulting from discrimination, like reduced physical activity and or social withdrawal, can exacerbate sleep disturbances, creating a self-perpetuating cycle of stress and poor sleep (20–22).

A growing body of research provides empirical evidence for an association between discrimination and sleep problems (14,23). Although much of this work has been cross-sectional and based on self-report measures of sleep, several studies—the earliest of which was facilitated by the honoree of this special issue—indicate that the association holds up longitudinally and with objective measures (24–31). However, few studies have examined subjective sleep problems or sleep duration (e.g., actigraphy-derived minutes of actual sleep from onset to wake time) as moderators of the association between discrimination and internalizing or externalizing symptoms, and those that have focused on adolescent samples (32,33). Fewer still have considered variability in sleep duration, defined as the within-person variance in minutes spent asleep across multiple adjacent nights of sleep assessment (33,34). This is a significant gap given that sleep problems and sleep variability are known to exacerbate the effects of a variety of adverse exposures (35–37), and show evidence of robust associations with mental and physical health (38–41). For example, more variability in sleep duration has been associated with higher urinary stress hormones (34), psychological distress (42), abdominal obesity (43), and inflammation (44). Furthermore, shorter habitual sleep and more variable sleep schedules have been associated with decreased task performance, more negative mood, and greater physiological wear and tear (45–52). Given established effects on cognition, affective mood states, emotion regulation, and related biological sequalae, it is not surprising that sleep problems and sleep variability have also been found to exacerbate associations between psychosocial stress and mental health problems (53,54).

Our focus in this manuscript is on actigraphic sleep duration variability, actigraphic sleep duration, and self-reported sleep problems. We focus on duration variability because of its emerging salience in the literature (55,56) and lack of consideration as a moderator of effects of discrimination on mental health outcomes. While other measures of variability have been considered (e.g., social jet lag, variability in timing), sleep duration variability is consistently associated with psychosocial and health outcomes and captures much of the meaningful variability in other measures of sleep variability (57). Sleep duration is an important consideration alongside sleep duration variability because of the correlation between the two and the need to consider the added predictive power of duration variability beyond duration alone. Underscoring their significance, duration and duration variability are also modifiable risk factors, and thus are potential targets for intervention at both individual and population levels. PSQI sleep problems adds breadth to our analyses and is important because of evidence suggesting that self-report measures of sleep capture important variability not captured by actigraphy (58,59). Although not the focus of this manuscript, results for additional actigraphy-assessed sleep measures (latency and efficiency) are provided in supplemental analyses.

Sleep problems and experiences of discrimination can also be considered as co-occurring risk factors and understood through the lens of a dual risk or multiplicate risk theoretical perspective. Experiences of multiple adverse conditions may be detrimental to well-being such that poor sleep may serve as a vulnerability that would exacerbate the effects of discrimination on mental health and well-being where the impact on health will be most evident (i.e., multiplicate) when both risk factors are present. This pattern of effects has growing empirical support in the context of sleep and discrimination experiences among adolescents (60–62).

A recent study found that adolescents with more variability in sleep duration had a stronger association between discrimination and internalizing and externalizing symptoms than adolescents who had less variable sleep (33). The same study also found that the association between discrimination and internalizing symptoms was exacerbated among adolescents with short duration or poor-quality sleep. Other studies focusing on discrimination and sleep among adolescents have also found evidence of multiplicative effects consistent with dual risk (63–66). Among diverse adolescents, longitudinal studies have reported that those who reported poor sleep quality and high levels of racial/ethnic discrimination have worse school engagement throughout high school (63), as well as greater internalizing symptoms (depressive symptoms, self-esteem) (65), whereas youth who report better sleep quality have attenuated relations between discrimination and well-being. Irregular sleep also served as a risk factor for poor academic outcomes and grades among adolescents who experienced higher levels of discrimination, compared to youth with more regular sleep (66). Additionally, longer and better-quality sleep has been identified as a protective factor for adolescents in the context of discrimination. Adolescents with longer sleep duration and less wake after sleep onset also engaged in greater coping skills and better well-being the next day after experiences of discrimination relative to those who had worse sleep (64).

Extending this new line of research, the objective of the current investigation was to examine subjective sleep problems, sleep duration, and sleep variability as moderating variables in the associations between discrimination and internalizing and externalizing mental health problems among adults. Moderators of risk, such as sleep, often represent a continuum of vulnerability to protective factor (67). Thus, our overarching hypothesis was that shorter and more variable sleep, and more global sleep problems, would exacerbate the association between discrimination and mental health problems. Otherwise put, our prediction based on prior work was that more healthy sleep would mitigate some of the adverse effects of discrimination.

## METHODS

### Participants

The analytic sample for the current investigation included 873 adults (499 women, 374 men). Participants were from two separate studies. Both studies included adolescents and their primary caregivers from small towns and semirural communities in the Southeastern United States (68,69). Recruitment letters were distributed in classrooms, and families contacted the laboratory about their interest in participating. Families were eligible if parents reported that their child did not have a diagnosed sleep disorder (e.g., sleep apnea) or learning disability. Our analyses focused on the adult parent sample from these studies. Participants from sample 1 consisted of 451 participants (270 women, 181 men) with data collected in 2011 and 2012. Sample 2 included 422 individuals (228 women, 194 men) whose data were collected from 2012 to 2013. Clustering of 873 individuals within 593 families was accounted for in all analyses (see analysis plan for details).

Sample 2 participants were slightly older (*M* = 42.56, SD = 6.33) and had higher family income-to-needs ratios (*M* = 2.47, SD = 1.29) than sample 1 participants (*M*_age_ = 38.77, SD = 7.16, *t*(854) = 8.20; *M*_INR_ = 1.57, SD = 1.00, *t*(762.73) = 10.97). There were no other significant differences across the two samples on study variables. Previous studies using these samples have not analyzed the two samples together, considered associations between discrimination and well-being, or considered the role of sleep in these associations.

The racial and ethnic composition of the sample (69% White or European American and 31% Black or African American) is largely representative of the communities that participants come from, which were 71% White and 23% Black. On average, women were 39.5 years old (SD = 6.53) and men were 42.1 years old (SD = 7.39). Individuals reported on family income and the number of other individuals in the household, which were used to derive income-to-needs ratios (INR), computed by dividing family income by the federal poverty threshold for that family size (US Department of Commerce; www.commerce.gov). Approximately 24% of individuals were living in poverty (INR < 1), 27% lived near the poverty line (1 < INR ≤ 2), 25% were lower middle class (2 < INR ≤ 3), 18% were middle class (3 < INR ≤ 4), and 6% were upper middle class (INR > 4).

### Procedure

The individual studies were approved by Auburn University’s Institutional Review Board, and participants provided their written consent for participation. Participants were asked towear actigraphs at home on their nondominant wrist for seven consecutive nights. To corroborate actigraphy data, each individual completed nightly sleep diary logs. Upon completion of sleep assessments, participants were emailed individual survey links (Qualtrics, Provo, UT) that were completed at home or at the campus laboratory.

### Measures

#### Discrimination

Participants reported on their experiences of discrimination using the modified 10-item Everyday Discrimination Scale (70,71). Individuals were asked how often in their day-to-day life they had experienced each type of discrimination (e.g., “you are treated with less respect than other people”; “people act as if they’re better than you are”; “you are threatened or harassed”). Items were rated on a four-point scale (1 = *often* to 4 = *never*). Responses were reverse coded and summed to create a total score, where higher scores indicated greater discrimination. Internal consistency was high for men (*α* = .92) and women (*α* = .89). Participants also reported whether they attributed their overall experiences to each of the following domains: language (< 5% of men and women; 5% White and 8% Black participants), gender (6% men, 12% women; 12% White and 21% Black participants), income (12% men, 15% women; 20% of Black and White participants), religion (< 5% of men and women; 9% White and 5% Black participants), race/ethnicity (11% men, 9% women; 8% White and 25% Black participants), body weight (4% men, 13% women; < 15% of Black and White participants), physical appearance (9% men, 8% women; < 15% of Black and White participants), clothing (8% husbands, 11% women; < 15% of Black and White participants), age (6% men; 7% of women; < 15% of Black and White participants), and whom they hang out with (< 7% of men and women; 9% White and 16% Black participants).

### Sleep

#### Actigraphy

Participants were asked to wear Octagonal Basic Motionlogger actigraphs (Ambulatory Monitoring, Ardsley, NY) at home while sleeping for 7 consecutive nights on their nondominant wrist. Sleep onset and wake times derived from the actigraph were corroborated with a sleep log. Data were scored in 1-minute epochs using zero crossing mode with the Action W2 software, and sleep was scored using Cole-Kripke algorithm (72). Sleep onset was determined from the first 3 minutes scored as sleep after reported bedtime, and wake time was determined from the last 5 consecutive minutes scored as sleep before reported wake time. The following sleep parameters were derived: a) sleep duration, the number of minutes scored as sleep between sleep onset and wake time; b) night-to-night variability in duration, computed using the coefficient of variation statistic of sleep duration; c) sleep efficiency, the percentage of epochs scored as sleep between sleep onset and wake time; and d) latency, the number of minutes from initial attempt at sleep in bed until sleep onset (73). Definitions of these sleep parameters are consistent with the manual for the actigraph and its associated software (Ambulatory Monitoring Inc, Ardsley, NY). Sleep duration, efficiency, and latency were averaged across available nights. Sleep data for participants with fewer than 5 out of 7 nights (27%) were treated as missing and handled statistically using full-information maximum likelihood (FIML) estimation (74). Reasons for missing data include exclusion of nights due to acute medication use for illness (e.g., cold, flu) and forgetting to wear the actigraph.

#### Subjective Sleep

Participants reported on their own sleep problems using the 19-item Pittsburgh Sleep Quality Index (PSQI; (50)). The PSQI assesses the frequency of multiple dimensions of sleep including duration, latency, quality, efficiency, and other sleep problems; items were rated on a four-point scale (0 = *not in the past month* to 3 = *three or more times a week*). A continuous global sleep problems score was derived (75). Scores in the current sample ranged from 0 to 21, with scores > 5 indicative of significant sleep problems (75). The measure has established psychometric properties for assessing sleep problems within a nonclinical sample (76). Significant sleep problems (i.e., scores > 5) were reported by 47% of men and 58% of women in the analytic sample. The PSQI had adequate internal consistency for men (*α* = .73) and women (*α* = .71).

### Internalizing and Externalizing Symptoms

Internalizing symptoms were assessed with measures of anxiety and depressive symptoms:

#### Symptoms of Anxiety

Participants reported on their anxiety using the 21-item Beck Anxiety Inventory (77), which has established psychometric properties in diverse populations. Participants reported on the extent to which they experienced common symptoms of anxiety (e.g., “feeling nervous,” “unable to relax”) over the past month using a four-point Likert scale (0 = *not at all* to 3 = s*everely*). Scores were summed to derive an anxiety symptoms score. The BAI demonstrated high internal consistency for men and women (*α* = .93 and *α* = .92, respectively). Scores in the mild-to-moderate clinical range (scores ranging from 10 to 18) were reported by 8% of men and 16% of women. A smaller percentage (6% men, 9% women) reported symptoms in the moderate-to-severe range (scores ranging from 19 to 29).

#### Symptoms of Depression

Men and women reported on their depressive symptoms using the 20-item Center for Epidemiologic Studies Depression Scale (CES-D; (53)). The scale assesses depression over the past week using items such as “I was bothered by things that don’t usually bother me” and “I felt sad.” Items are rated using a four-point scale (0 = *rarely* to 3 = *most or all of the time*). An item assessing sleep problems was removed, and scores were summed to create an overall depressive symptoms score. The CES-D demonstrated good reliability (men: *α* = .88; women: *α* = .89). Analyses indicate that 16% of men and 22% of women surpassed the clinical cutoff for depression (score ≥16).

#### Externalizing Symptoms

The hostility scale of the SCL-90 was used to assess externalizing symptoms. Participants reported on their feelings of hostility over the past 2 weeks using the Symptoms Checklist-90 Revised (SCL-90R; (54)). Hostility was assessed using six items (e.g., “feeling easily annoyed or irritated,” “having urges to break or smash things”) that assessed how bothered they were using a 5-point scale (0 = *not at all* to 4 = *extremely*). Scores were summed, and higher scores indicated greater externalizing symptoms. The hostility scale of the SCL-90R demonstrated good reliability for both men (*α* = .81) and women (*α* = .77).

### Controls

Participant age, gender (0 = male, 1 = female), race (0 = White; 1 = Black/AA), cohabitation status (0 = not living with a partner, 1 = living with a partner), study (0 = study 1, 1 = study 2), and family income-to-needs ratio (described above) were used as covariates in all models.

### Analysis Plan

To reduce outlier effects, scores that exceeded 3 SDs from the mean were winsorized and recoded to the highest or lowest value within 3 SD (*n* = 47; (46)). None of the study variables were skewed (skewness statistics < 2). A series of regression models were fit using Mplus 8.7 (78). To account for the non-independence of married or cohabiting couples, all models utilized the “type = complex” specification in Mplus to provide accurate estimates of the standard errors in the nested dataset (78). FIML was used to handle missing data, which produces less biased estimates and fewer type I errors than other methods of handling missing data (Raykov, 2005). Rates of missing data were relatively low (11%–15%) among most study variables and moderate for actigraphy (29%); rates are within acceptable guidelines for the use of FIML (79).

Separate models were fit for each sleep index and for each mental health outcome. Covariates, discrimination, and the relevant sleep parameter(s) were entered in the first step of all models. To ascertain the unique effects of variability in sleep duration, sleep duration was included as an additional covariate in models that examined variability. In the second step, all variables were mean-centered, and two-way interactions between discrimination and the relevant sleep index were added to the models. Finally, three-way interactions with race, gender, and INR were added individually to each model, along with all lower-order two-way interactions between discrimination, sleep, and race/gender/INR. Exogenous variables were allowed to covary, and models were fully saturated; thus, model fit statistics are not reported. Statistically significant (*p* < .05) interactions were plotted at high and low levels (±1 SD) of discrimination and sleep. Simple slope analyses were conducted to examine whether discrimination slopes were significantly different from zero at varying levels of sleep. The term “predict” is used as a statistical term and is not indicative of causality.

## RESULTS

### Preliminary Analyses

Means, standard deviations, and correlations for study variables are presented in Table 1. Discrimination was associated with more subjective sleep problems, anxiety, depression, and externalizing symptoms. Participants who slept longer had less variable sleep durations and reported fewer subjective sleep problems. Greater variability in sleep and more subjective sleep problems (but not sleep duration) were associated with greater anxiety, depression, and externalizing symptoms. Anxiety, depression, and externalizing symptoms were all moderately correlated.

Independent samples *t* tests revealed no significant gender or race differences in discrimination (Table S1, Supplemental Digital Content, http://links.lww.com/PSYMED/B17). Women had longer sleep, less variability in sleep, greater subjective sleep problems, more anxiety, and more depression than men. Black participants had shorter sleep, more variable sleep, and lower levels of anxiety and externalizing symptoms compared to White participants.

### Sleep as a Moderator of Associations Between Discrimination and Mental Health Problems

#### Actigraphy Indices

Initial models examined discrimination, sleep duration, and all covariates as predictors of each mental health outcome; the discrimination by sleep duration interaction was entered in a second step. Neither the sleep duration direct effect nor the interaction term was significant in predicting any mental health outcome.

**FIGURE 1.**
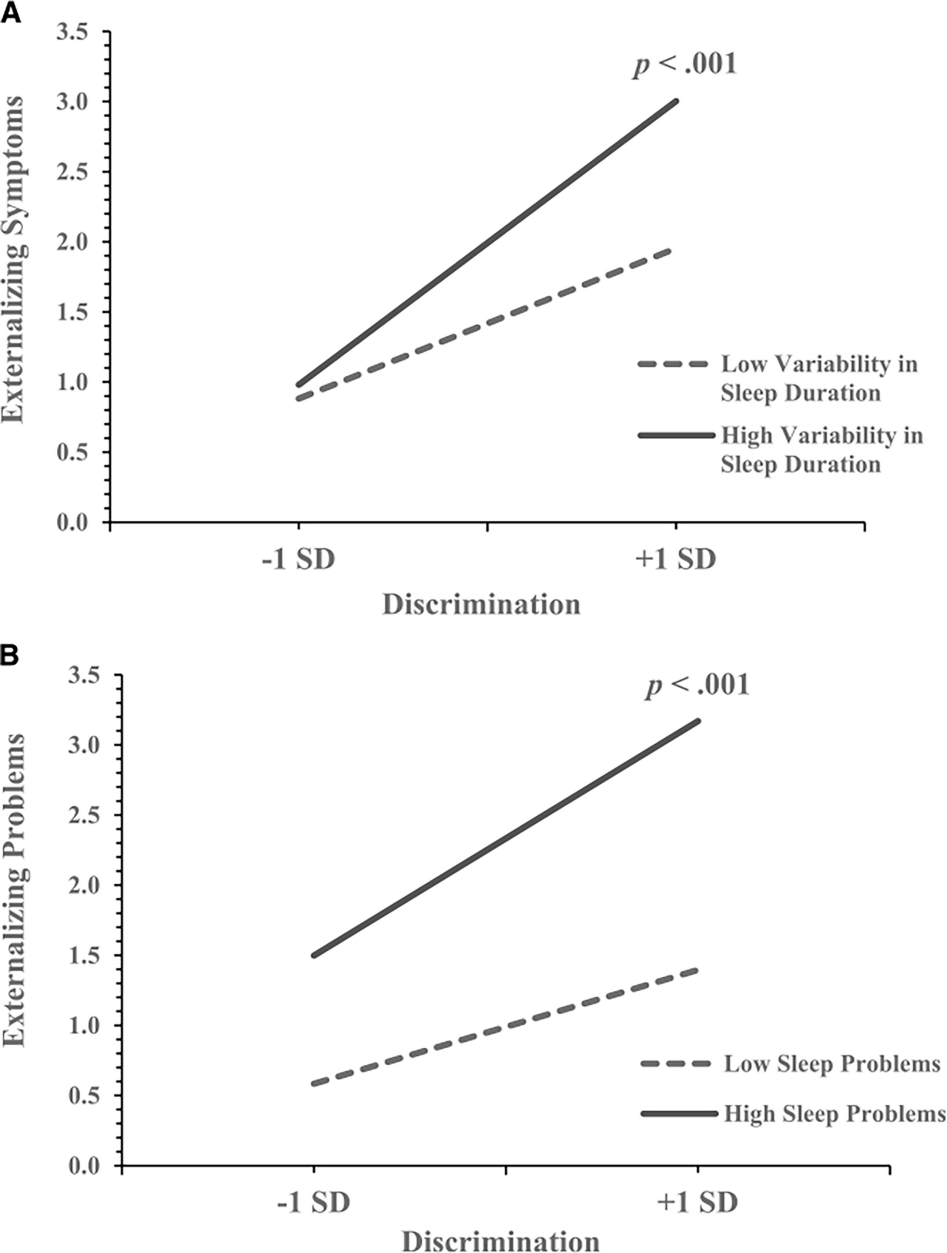
Associations between discrimination and externalizing symptoms at low (−1 SD) and high (+1 SD) levels of variability in sleep duration (A) and subjective sleep problems (B). *p* Values indicate the statistical significance of each slope.

Next, we added variability in sleep duration to the models (Table 2). After accounting for the covariates, the direct effect models revealed that more experiences of discrimination and more variability in sleep duration were each associated with greater anxiety, depression, and externalizing symptoms. To test moderation effects, the interaction between discrimination and variability in sleep duration was added to the models. Discrimination interacted with variability in sleep duration to predict externalizing symptoms (Figure 1A). Simple slopes analysis indicated that the positive association between discrimination and externalizing symptoms was stronger for participants with high variability in duration (*B* = 0.19, SE = 0.02) compared to those with low variability in duration (*B* = 0.11, SE = 0.03). Participants with both high levels of discrimination and high variability in sleep duration reported the highest levels of externalizing symptoms in the sample (predicted *M* = 3.00), a difference of 0.47 SD higher compared to those with high discrimination but low variability in sleep duration (predicted *M* = 1.95). Participants with low levels of discrimination reported relatively low levels of externalizing symptoms with either high (predicted *M* = 0.98) or low (predicted *M* = 0.88) variability in sleep.

##### Consideration of Additional Sleep Actigraphy Measures

Two additional sleep actigraphy parameters were also considered: latency and efficiency. Latency was a significant direct predictor of anxiety (*B* = 0.10, SE = 0.03, *β* = .15, *p* = .001), but did not moderate the association between discrimination and mental health outcomes. No direct effects of efficiency on mental health outcomes were evident, and the association between discrimination and externalizing was not moderated by efficiency in the expected direction. However, an interaction was evident (*B* = 0.01, SE = 0.00, *β* = .07, *p* = .049). Post hoc probing of this interaction revealed similar, significant associations between discrimination and externalizing at low (*B* = 0.12, SE = 0.02) and high (*B* = 0.19, SE = 0.03) levels of efficiency (both *p* values < .001). When these additional sleep actigraphy measures were added to models examining duration and variability in duration, the substantive findings and patterns of inference reported above were equivalent/unchanged.

#### Subjective Sleep Problems

After accounting for covariates and the effect of discrimination, there was a significant direct effect of subjective sleep problems on each mental health outcome, such that greater sleep problems were associated with higher levels of anxiety, depression, and externalizing problems (Table S2, Supplemental Digital Content, http://links.lww.com/PSYMED/B17). Further, discrimination interacted with subjective sleep problems to predict anxiety and externalizing symptoms. Simple slopes testing revealed that the positive association between discrimination and anxiety was stronger and significant for participants with greater subjective sleep problems (*B* = 0.43, SE = 0.08) and marginally significant for those with fewer subjective sleep problems (*B* = 0.13, SE = 0.07; Figure S1, Supplemental Digital Content, http://links.lww.com/PSYMED/B17). Among participants who perceived high levels of discrimination, those with greater subjective sleep problems reported higher levels of anxiety (predicted *M* = 10.27) than those with fewer sleep problems (predicted *M* = 3.04), a difference of 1.05 SD. Participants with high levels of subjective sleep problems and higher perceived discrimination also reported greater anxiety than their counterparts with lower perceived discrimination (predicted *M* = 5.90), a 0.63 SD difference. Participants with low perceived discrimination and few subjective sleep problems reported the lowest levels of anxiety in the sample (predicted *M* = 1.72), whereas the highest levels of anxiety were observed for those with high levels of perceived discrimination and greater subjective sleep problems.

The interaction between discrimination and sleep problems predicting externalizing symptoms revealed a similar pattern (Figure 1B). The positive association between discrimination and externalizing symptoms was stronger for participants with greater subjective sleep problems (*B* = 0.16, SE = 0.03) compared to those with fewer subjective sleep problems (*B* = 0.08, SE = 0.03). Among participants who perceived high levels of discrimination, those with greater subjective sleep problems reported greater externalizing symptoms (predicted *M* = 3.17) than their counterparts with fewer sleep problems (predicted *M* = 1.40), a difference of 0.79 SD. Participants who reported high levels of subjective sleep problems and high levels of discrimination also reported greater externalizing symptoms than those with sleep problems and low levels of discrimination (predicted *M* = 1.50), a difference of 0.75 SD. Participants with fewer subjective sleep problems and lower perceived discrimination reported the lowest levels of externalizing symptoms (predicted *M* = 0.58).

To ascertain the unique effects of subjective and objective sleep parameters, we additionally considered actigraphy indices and self-reported sleep problems in the same model. The direct effect of subjective sleep problems remained a significant predictor of anxiety (*B* = 0.72, SE = 0.07, *β* = .40, *p* < .001), depression (*B* = 0.77, SE = 0.09, *β* = .36, *p* < .001), and externalizing symptoms (*B* = 0.17, SE = 0.03, *β* = .29, *p* < .001). Additionally, the discrimination by PSQI interaction remained a significant predictor of anxiety (*B* = 0.04, SE = 0.01, *β* = .11, *p* = .005) and externalizing symptoms (*B* = 0.01, SE = 0.01, *β* = .08, *p* = .035), and the discrimination by variability in duration interaction remained a significant predictor of externalizing symptoms (*B* = 0.04, SE = 0.02, *β* = .09, *p* = .047).

### Three-Way Interactions With Race, Gender, and Socioeconomic Status

We additionally explored interactions between discrimination, each measure of sleep individually, and demographic characteristics. No interactions with socioeconomic status (INR) were significant. Additionally, no race or gender interactions were evident in models considering actigraphy-assessed measures of sleep. However, the discrimination by subjective sleep problems by race interaction term significantly predicted anxiety, *B* = −0.07, SE = 0.03, *β* = −.09, *p* = .011. Simple slopes analysis revealed that the positive association between discrimination and anxiety was significant for White and Black participants with greater subjective sleep problems and Black participants with fewer subjective sleep problems, but not White participants with fewer sleep problems, who had relatively low levels of anxiety regardless of discrimination (Figure S2, Supplemental Digital Content, http://links.lww.com/PSYMED/B17). No other three-way interactions with race were evident.

Additionally, the discrimination by subjective sleep problems by gender interaction term significantly predicted depression, *B* = −0.06, SE = 0.03, *β* = −.07, *p* = .042. Simple slopes analysis indicated a significant association between discrimination and higher levels of depression for men and women with greater subjective sleep problems and women with fewer sleep problems (Figure S3, http://links.lww.com/PSYMED/B17). The positive association was relatively weaker and marginally significant for men with few subjective sleep problems, who had relatively low levels of depression regardless of discrimination. No other three-way interactions with gender were evident.

## DISCUSSION

Accumulating evidence suggests that experiences of discrimination have a marked impact on internalizing and externalizing symptoms, and recent studies conducted with adolescent samples suggest that sleep may play an important role in moderating this association (33,64). Adding to this research and extending it to the adult age range, the current study examined three measures of sleep (duration, variability in duration, and global sleep problems), as moderating variables in the association between discrimination and mental health problems (depression, anxiety, externalizing symptoms). Consistent with a multiplicative risk perspective (60,61), results indicated that among those with more sleep duration variability or more global sleep problems (but not shorter sleep duration), discrimination was more strongly associated with externalizing symptoms. Results were less consistent for measures of internalizing symptoms (depression, anxiety); actigraphy-assessed sleep measures did not significantly moderate the effects of discrimination on depression or anxiety, but the association between discrimination and anxiety was greater among those with more self-reported global sleep problems.

Prior studies of adolescents have reported an interaction between discrimination and sleep such that those with poor sleep and more experiences of discrimination had more internalizing and externalizing symptoms, engaged in more negative coping strategies, and had lower levels of academic engagement (32,63–65). To our knowledge, few if any studies to date have considered the interactive effects of sleep and discrimination as predictors of internalizing and externalizing symptoms among adults. The result that sleep parameters moderate the association between discrimination and mental health problems among adults is thus an important extension of prior work and suggests that further examination of sleep parameters in the contexts of discrimination and associated stressors is warranted.

Results indicated that the moderating role of actigraphy-assessed sleep was more consistent for externalizing than for internalizing problems. More research is needed to determine why this might be the case. One possibility is that, when sleep problems are greater, the emotional and cognitive resources to regulate anger and negative emotion could be compromised leading to greater overall increases in measures of externalizing symptoms. This interpretation aligns with other literatures documenting the cognitive burden of discrimination and is sequelae (80,81). Another possibility complementing this interpretation relates to established literature linking sleep problems to violence, aggression, and hostility. These studies emphasize the role of sleep problems in the dysregulated functioning of prefrontal cortical brain regions and hypothalamic-pituitary-adrenal axis pathways leading to compromised self-regulatory capacities and increased chances of aggression in response to challenge and stress (82–85).

Results also add importantly to an emerging literature on the role of sleep variability in psychosocial stress and health outcomes. Prior work has shown that actigraphy-assessed sleep duration variability is associated with greater perceived stress among young adults (42) and higher levels of urinary stress hormones (34). Recent work has also found evidence that sleep variability moderates associations between discrimination and mental health problems among adolescents (33). Our study extends this work by showing that sleep variability moderates the association between discrimination and mental health problems among adults. Otherwise put, the findings suggest that sleep moderation effects, which have been documented among adolescents experiencing discrimination, appear to persist into adulthood. That findings for sleep duration variability were stronger than for duration itself replicates previous findings with adolescents (86–88) and underscores the significance of duration variability as a consequential predictor (89). Variability in duration, and similar variables, may capture aspects of circadian and behavioral rhythms that may not be reflected in other actigraphic measures such as mean duration (57). Nonetheless, we do not wish to overemphasize the null findings for sleep duration, which could relate to the idiosyncrasies of this particular sample. We expect that as literature grows, both duration and duration variability will be important predictors of stress responses and health outcomes.

In the context of research examining sleep as a moderator of associations between discrimination and health, it is vital to note that, although studies focusing on individual-level moderators are invaluable in understanding personal resilience and coping, they should not be misconstrued to imply that systemic injustices can be rectified through individual-level interventions alone. This misinterpretation can overshadow the need for broader societal reforms and can be detrimental to positive social change. Therefore, research must balance the significance of personal factors with the recognition that health disparities are fundamentally rooted in systemic issues. Other lines of research such as exploring the sources of discrimination (90) or elucidating the role of discrimination as a mediator of health disparities (91,92) are therefore essential complements to this research.

For all actigraphy-assessed measures of sleep, the reported findings were consistent across race, gender, and socioeconomic categories. However, analyses for self-reported global sleep problems suggested a race moderation effect for anxiety (but not depression or externalizing symptoms) and a gender moderation effect for depression (but not anxiety or externalizing symptoms). Specifically, the moderating role of sleep problems in the association between discrimination and anxiety was found to be greater for White than Black participants, and greater for men than for women. That is, White adults and men had greater protection from better sleep (i.e., less sleep problems) than Black adults or women. More research will be needed to confirm these group differences and examine explanations. Possibilities include race or gender differences in types of discrimination experienced or group differences in assessment of self-report measures of sleep. Also, important to note is that, although our focus in this manuscript was on discrimination and sleep as predictors of mental health problems, mental health is also an established predictor of sleep. Bidirectional associations and the presence of mutually reinforcing linkages between sleep and mental health problems are therefore an important consideration. Elucidation of moderators of associations in either direction—as a means of breaking negative feedback loops that lead to clinically significant mental disorders or supporting positive feedback loops that optimize functioning—are of particular significance and underscore the salience of this line of research.

Some limitations are important to note. First, our measures of internalizing and externalizing problems and discrimination, although well validated and established, are based on self-report measures and thus are subject to potential reporting bias. This opens the possibility that associations between discrimination and mental health problems could be influenced by third variables (e.g., personality factors affecting reports of both discrimination and mental health). Previous studies using experimental and longitudinal methods help to mitigate this concern by providing evidence of a directional association between discrimination and mental health (8,9). However, future research using alternative measures and methods to assess mental health or discrimination (e.g., clinician assessed measures of mental health or areas-level indexes of discrimination) will be useful to advance this line of research. Furthermore, although the discrimination measure used in this study is well established and widely used, it will be useful for future work to consider a broader range of discrimination-related stressors. Measures of vicarious discrimination and vigilance, for example, will be important candidate measures.

Second, all of our data were collected between 2011 and 2013 and should be interpreted accordingly. It will be useful for future research to consider more recently administered measures of discrimination or examine whether levels or correlates of discrimination may have changed (20,93). Third, although some of our sleep parameters were assessed objectively using actigraphy, sleep problems were assessed via an established survey measure. Our findings relating to self-reported sleep problems could therefore be affected by reporting bias. However, consistency in the direction of reported effects across self-report and objective sleep measures partially allays these concerns. Nonetheless, other measures of sleep (e.g., from self-reported diary measures) will be useful for future research to examine. Fourth, although our study is unique in examining the combined effects of discrimination and sleep on internalizing and externalizing symptoms, we were not able to consider the role of other health or health behavior measures such as BMI or cigarette smoking as mechanistic pathways or control variables in the reported associations. Future research will therefore be useful to provide insight into the role of these measures.

Lastly, although our study had a relatively large sample size, was well powered to address the hypotheses, and allowed for consideration of results by race, gender, and socioeconomic status, our sample was country and region specific (Southeastern United States) and focused on a sample of adults within a specific age range. Additional research will therefore be needed to extend our findings to other contexts or demographic groups. Comparative studies will be useful to advance knowledge on how the role of sleep may differ across contexts (e.g., national, urban, etc.). Building from a large literature documenting a robust association between discrimination and mental health problems, the reported findings provide new evidence that sleep is an important modifiable factor that may help to mitigate the adverse mental health consequences of discrimination among adults.

## Supplementary Material

Supplementary_FILES

## Figures and Tables

**TABLE 1.** Correlations and Descriptive Statistics

	1	2	3	4	5	6	7	8	9	10	11	12
1. Gender (female)	—											
2. Race (Black)	.10**	—										
3. Cohabitating	−.26***	−.26***	—									
4. Age (y)	−.19***	−.21***	.19***	—								
5. Income-to-needs ratio	−.08*	−.33***	.29***	.30***	—							
6. Discrimination	−.04	.02	.05	.02	−.11**	—						
7. Sleep duration (min)	.16***	−.34***	.06	−.06	.20***	−.03	—					
8. Sleep duration variability	−.10*	.20***	−.04	−.07	−.12**	.06	−.55***	—				
9. PSQI (sleep problems)	.09*	−.01	−.11*	.06	−.18**	.24***	−.10***	.23***	—			
10. Anxiety	.16***	−.11**	−.11*	.06	−.11*	.32***	.00	.10*	.49***	—		
11. Depression	.14**	.02	−.14**	−.05	−.21***	.37***	−.02	.08*	.46***	.58***	—	
12. Externalizing	.06	−.13***	.01	−.05	−.08*	.32***	.04	.10*	.37***	.52***	.57***	—
Mean	.57	.31	.82	40.6	2.03	14.88	383.8	.18	6.58	5.60	9.03	1.81
Standard deviation	—	—	—	7.27	1.24	5.13	68.9	.10	3.87	7.23	8.46	2.61

PSQI = Pittsburgh Sleep Quality Index of Global Sleep Problems.

Gender was coded as 0 = male, 1 = female. Race was coded as 0 = White, 1 = Black/African American. Cohabitating was coded as 0 = not cohabitating, 1 = married or living with a partner.

* *p* < .05.

** *p* < .01.

*** *p* < .001.

**TABLE 2.** Discrimination, Actigraphy Sleep Parameters, and Their Interaction Predicting Mental Health Problems

	Anxiety				Depression				Externalizing Problems			
			
	*B*	SE	*β*	*R* ^2^	*B*	SE	*β*	*R* ^2^	*B*	SE	*β*	*R* ^2^
Study cohort	−0.24	0.58	−.02		−0.27	0.63	−.02		0.16	0.18	.03	
Gender (female)	2.69	0.45	.19***		2.05	0.55	.12***		0.43	0.14	.10**	
Race (Black)	−2.66	0.69	−.18***		−1.38	0.83	−.08		−0.91	0.23	−.19***	
Age	0.12	0.04	.12**		0.01	0.05	.01		−0.01	0.01	−.05	
Cohabitation	−1.71	0.81	−.10*		−1.88	0.96	−.09		−0.14	0.22	−.02	
Inc-to-needs	−0.70	0.30	−.13*		−1.07	0.34	−.16**		−0.08	0.09	−.04	
Discrimination	0.42	0.06	.31***		0.59	0.06	.36***		0.15	0.02	.34***	
Duration	0.01	0.01	.05		0.00	0.00	.04		0.00	0.00	.06	
Variability in duration	1.26	0.36	.18***		0.91	0.41	.11*		0.39	0.12	.17**	
				20.3%				20.1%				18.2%
Discrimination by variability in duration	0.04	0.06	.03		0.01	0.06	.01		0.04	0.02	.10*	
				20.4%				20.1%				19.2%

Inc-to-needs = income-to-needs ratio.

Reported coefficients are from the step of entry (interaction term is added in second step). Gender was coded as 0 = male, 1 = female. Race was coded as 0 = White, 1 = Black/African American. Cohabitation was coded as 0 = not cohabitating, 1 = married or living with a partner.

* *p* < .05.

** *p* < .01.

*** *p* < .001.

## Data Availability

Data will be made available after completion of each longitudinal study in accordance with National Institutes of Health data sharing guidelines.

## Abbreviations:

BAI: Beck Anxiety Inventory
CES-D: Center for Epidemiologic Studies Depression Scale
FIML: full-information maximum likelihood
INR: income-to-needs ratio
PSQI: Pittsburgh Sleep Quality Index
SCL-90R: Symptoms Checklist-90 Revised
