# Supplementary material for: Do Sleep Problems Exacerbate the Mental Health Consequences of Discrimination Among Adults?
Source: Psychosom Med. Author manuscript; Available in PMC 2024 Dec 6. (PMC11622914; doi:10.1097/PSY.0000000000001305)
Supplement: Supplementary_FILES [file NIHMS2019043-supplement-Supplementary_FILES.pdf]

Supplemental Materials

Table S1

Gender and Race Differences in Discrimination, Sleep, and Mental Health Problems

	White		Black			Men		Women		
	M	(*SD*)	*M*	(*SD*)	*t*-value	*M*	(*SD*)	*M*	(*SD*)	*t*-value
Discrimination	14.81	(4.83)	15.00	(5.59)	-0.44	15.10	(5.20)	14.70	(5.01)	1.06
Sleep duration (minutes)	399.74	(62.76)	349.35	(64.75)	**8.17*****	370.89	(70.32)	393.53	(66.14)	**-4.11*****
Variability in sleep duration	0.16	(0.09)	0.21	(0.11)	**-4.35*****	0.19	(0.11)	0.17	(0.09)	**2.46***
Sleep Problems	6.51	(3.92)	6.42	(3.50)	0.29	6.12	(3.93)	6.88	(3.74)	**-2.67****
Anxiety	6.06	(7.17)	4.54	(6.06)	**2.88****	4.15	(6.31)	6.44	(7.15)	**-4.69*****
Depression	8.85	(8.49)	9.21	(7.76)	-0.52	7.69	(7.71)	9.88	(8.63)	**-3.70*****
Externalizing	1.94	(2.30)	1.27	(2.12)	**3.77*****	1.55	(2.17)	1.85	(2.30)	-1.83

Note. * $p < .05$. ** $p < .01$. *** $p < .001$.

Table S2

Discrimination, Subjective Sleep Problems, and their Interaction Predicting Mental Health Problems

	Anxiety				Depression				Externalizing			
	B	*SE*	β	R^2	*B*	*SE*	β	R^2	*B*	*SE*	β	R^2
Study cohort	-0.06	0.49	-.00		-0.14	0.55	-.01		0.19	0.16	.04	
Gender (female)	2.02	0.39	.15***		1.48	0.51	.09**		0.26	0.14	.06	
Race (Black)	-1.71	0.54	-.12**		-0.68	0.72	-.04		-0.71	0.19	-.15***	
Age	0.11	0.04	.11**		0.01	0.04	.01		-0.02	0.01	-.05	
Cohabitation	-1.10	0.76	-.06		-1.32	0.88	-.06		0.01	0.22	.00	
Inc-to-needs	-0.29	0.27	-.05		-0.69	0.31	-.10*		0.02	0.09	.01	
Discrimination	0.29	0.05	.22***		0.46	0.06	.28***		0.12	0.02	.28***	
Sleep problems	0.77	0.07	.43***		0.79	0.08	.36***		0.18	0.02	.32***	
				34.4%				31.4%				25.0%
Disc. x Sleep problems	0.04	0.01	.11**		0.02	0.02	0.05		0.01	0.00	.09*	
				35.6%				31.6%				25.8%

Note. Reported coefficients are from the step of entry. Sex was coded as 0 = male, 1 = female. Race was coded as 0 = White, 1 = Black/African American. Cohabitation was coded as 0 = not cohabitating, 1 = married or living with a partner. Inc-to-needs = Income-to-needs ratio. Disc. = Discrimination.

* $p < .05$, ** $p < .01$, *** $p < .001$

Figure S1

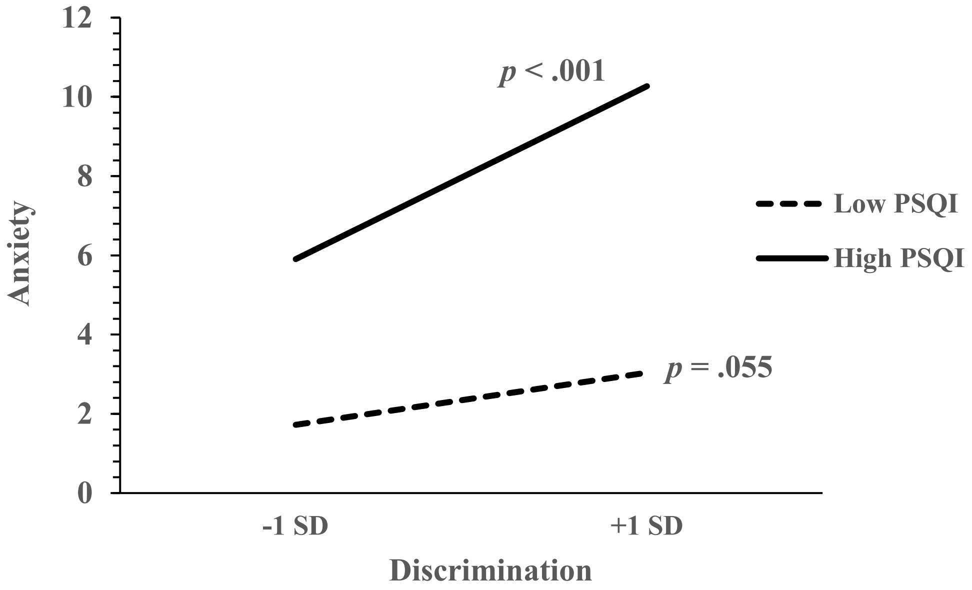

Associations between discrimination and anxiety at lower (-1 SD) and higher (+1 SD) levels of subjective sleep problems. PSQI = Pittsburgh Sleep Quality Index of sleep problems.

Figure S2

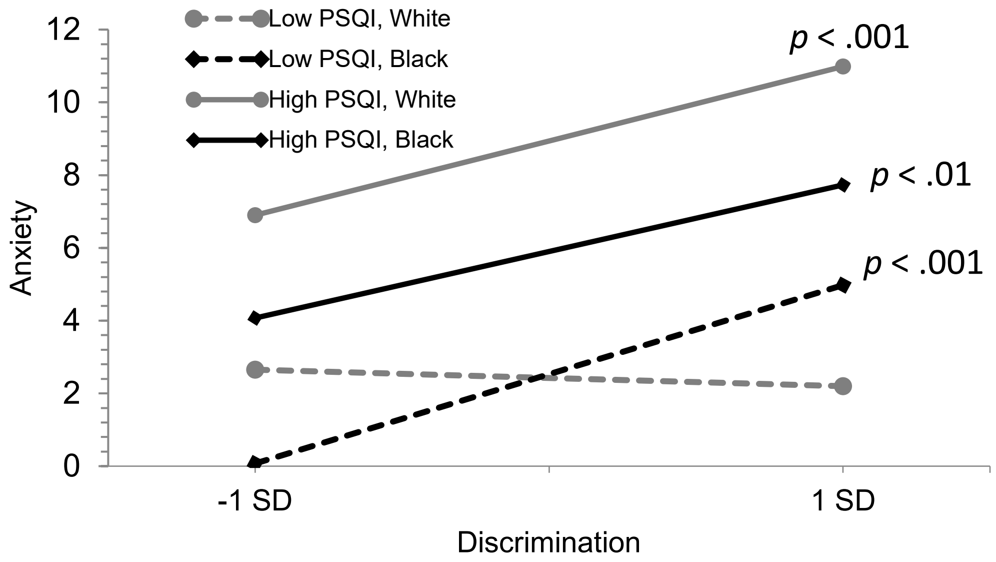

Associations between discrimination and anxiety at lower (-1 SD) and higher (+1 SD) levels of subjective sleep problems among Black and White adults. PSQI = Pittsburgh Sleep Quality Index of sleep problems.

Figure S3

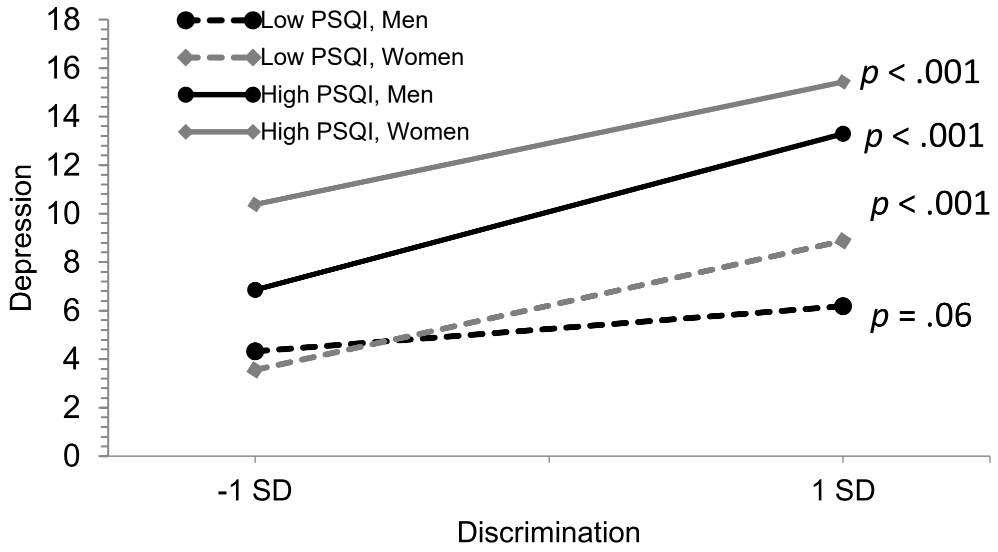

Associations between discrimination and depression at lower (-1 SD) and higher (+1 SD) levels of subjective sleep problems among men and women. PSQI = Pittsburgh Sleep Quality Index of sleep problems.
